# Similarities in Structure and Function of UDP-Glycosyltransferase Homologs from Human and Plants

**DOI:** 10.3390/ijms25052782

**Published:** 2024-02-28

**Authors:** Mary Caroline L. Lethe, Vincent Paris, Xiaoqiang Wang, Clement T. Y. Chan

**Affiliations:** 1 Department of Biomedical Engineering, College of Engineering, University of North Texas, 3940 N Elm Street, Denton, TX 76207, USAvincent.paris@unt.edu (V.P.); 2 Department of Biological Sciences, College of Science, University of North Texas, 1155 Union Circle #305220, Denton, TX 76203, USA; xiaoqiang.wang@unt.edu; 3 BioDiscovery Institute, University of North Texas, 1155 Union Circle #305220, Denton, TX 76203, USA

**Keywords:** uridine diphosphate glycosyltransferases, glycosylation, substrate specificity, UGT-related diseases

## Abstract

The uridine diphosphate glycosyltransferase (UGT) superfamily plays a key role in the metabolism of xenobiotics and metabolic wastes, which is essential for detoxifying those species. Over the last several decades, a huge effort has been put into studying human and mammalian UGT homologs, but family members in other organisms have been explored much less. Potentially, other UGT homologs can have desirable substrate specificity and biological activities that can be harnessed for detoxification in various medical settings. In this review article, we take a plant UGT homology, UGT71G1, and compare its structural and biochemical properties with the human homologs. These comparisons suggest that even though mammalian and plant UGTs are functional in different environments, they may support similar biochemical activities based on their protein structure and function. The known biological functions of these homologs are discussed so as to provide insights into the use of UGT homologs from other organisms for addressing human diseases related to UGTs.

## 1. Introduction

**Figure 1:**
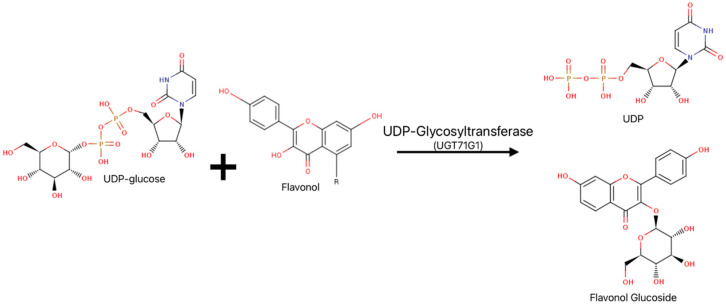
General UGT reaction scheme represented by UGT71G1. Among plant UGT homologs, UDP-glucose is the preferred UDP sugar donor; flavonols are natural plant metabolites that serve as acceptors. Functional groups that involve nitrogen are shown in blue and those with oxygen are shown in red.

Glycosylation is an essential metabolic processes that facilitates the detoxification, storage, or excretion of xenobiotics and endogenous substrates. Significant to this process are uridine diphosphate glycosyltransferases (UGTs), which catalyze the transfer of sugar moieties from uridine diphosphate (UDP) sugar donors to various acceptor molecules (Figure 1) [1]. Plant UGTs are classified into the glycosyltransferase family 1 (GT1), in which all present a GT-B fold consisting of N-terminal and C-terminal Rossman-like domains involved in the binding of a sugar donor and acceptor [2,3].

UGT71G1 (Figure 1) is a plant UGT that utilizes UDP-glucose as a donor and flavonoids and triterpenes as acceptors [4]. It was the first plant UGT to be structurally analyzed, and the detailed interactions uncovered between the enzyme and its substrates provided a basis for understanding substrate recognition and regiospecificity within the plant UGT superfamily [1]. Glycosylation is often on O-atoms, but some UGTs also perform N-, S-, or C-glycosylation [3]. Sugar donors recognized by UGTs include, but are not limited to, UDP-glucose, UDP-glucuronic acid, UDP-galactose, and UDP-xylose, with the use of UDP-glucuronic acid being the most documented in animal UGTs and UDP-glucose in plant UGTs [5,6].

Glucuronidation is a process in phase II metabolism where the glycosylation of xenobiotics increases solubility and aids in their elimination. In both humans and plants, UGT is vital to the detoxification and regulation of bioactive substances and has been investigated for its roles in the metabolism of specific substrates. Therefore, the dysfunction or absence of UGTs can have major consequences related to the build-up of metabolites, which would typically be glycosylated. Several metabolic disorders caused by UGT dysfunction have been described, as well as the roles of UGTs in steroid regulation and xenobiotic elimination regarding human health. Some of these cases are described in this article.

Through this article, we aim to generate new insights into tackling human diseases related to UGT dysfunction. Here, we have compared the structure and function of UGT homologs from humans and plants, evaluating the feasibility of using homologs from other organisms to complement human UGT activities. We have also described some related diseases and discussed how plant UGT homologs may provide benefits to these situations.

## 2. Classification of UGT Homologs

The defining characteristic of UGTs is a highly conserved consensus sequence or “UDP-glycosyltransferase signature” of about 40–50 amino acids in length (PROSITE accession number PS00375). This motif is usually in the C-terminal domain and involved in the binding of the protein to the UDP moiety of the sugar nucleotide [4]. In 1991, an official UGT gene nomenclature system was proposed based on amino acid homology and the presence of a consensus sequence [7].

**Figure 2:**
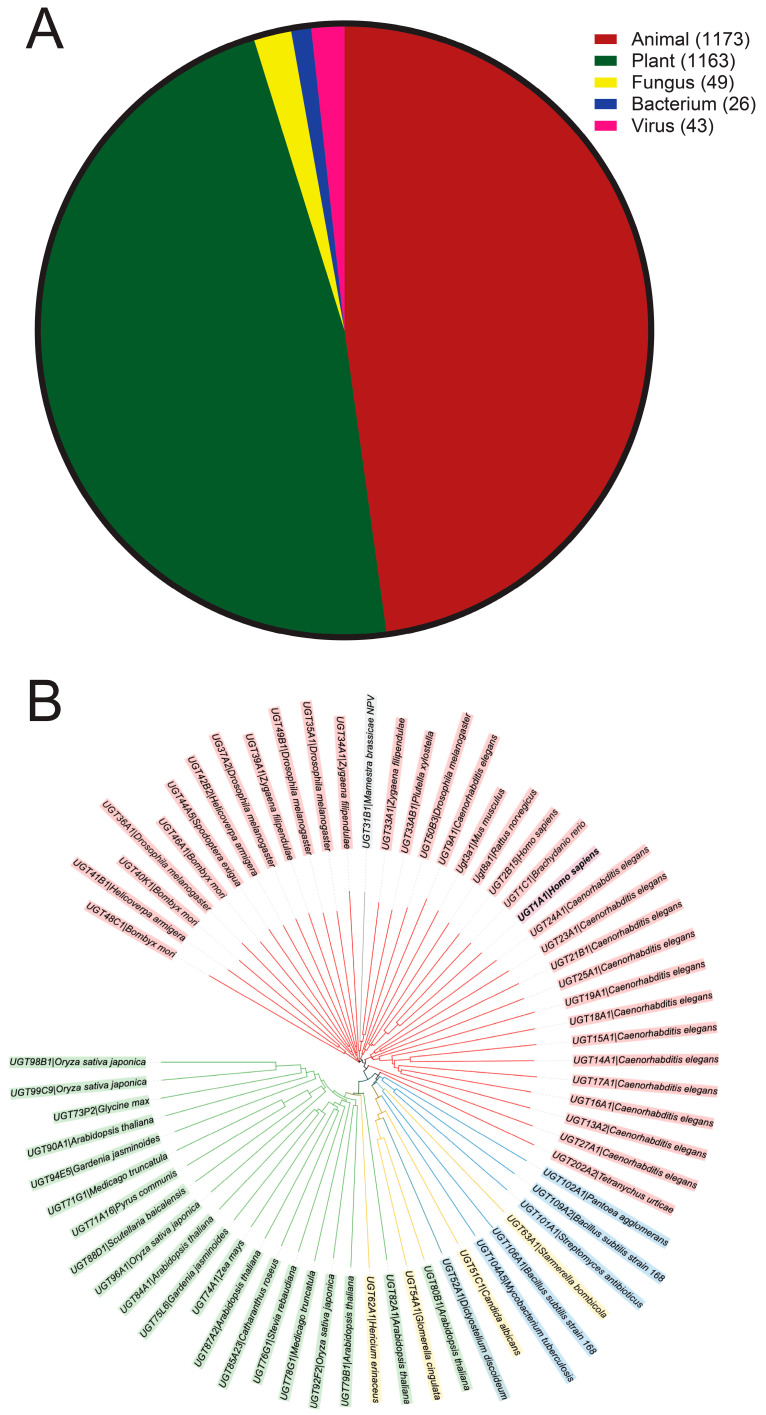
UGT homologs from different kingdoms. (**A**) Identified UGT homologs from different kingdoms. Information was collected from the database of the UGT Nomenclature Committee. The number of UGT homologs from each kingdom is shown. (**B**) Non-exhaustive phylogenic tree of UGTs with colors representing kingdoms. A range of UGT homologs were arbitrarily selected from each kingdom for this analysis, aiming to demonstrate the homology of UGT homologs from different kingdoms. Red/pink: animals; green: plants; blue: bacteria; yellow: fungi; gray: viruses; teal: other (amoeba). The tree structure was generated from UniProt using the Clustal Omega program (http://www.clustal.org/omega/; accessed on 8 November 2023) and modified with iTOL. UGT data are from UGT Nomenclature Committee 2023 UGT names files and UniProt.

UGT families are organized by phylogeny, with families 1 to 2 being humans and 71 to 100 being plant species (Figure 2). These UGT families share at least 40% homology, and are further divided into subfamilies sharing at least 60% homology [4]. UGT71G1 (EC 2.4.1.91) is in family 71 and subfamily G, and the final number indicates an individual gene. The UGT Nomenclature Committee [7] has compiled a list that currently contains over 80 UGT71 protein and pseudogene entries, with UGT71G1 being the only one in its subfamily; over 40,000 UDP-glycosyltransferases have been documented and about 400 UGTs have been verified at the protein level [8].

## 3. Substrate Recognition and Biological Activities of UGTs

**Figure 3:**
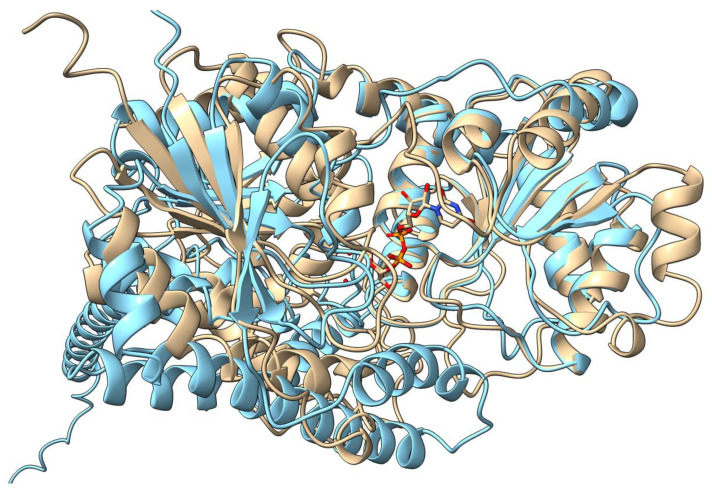
Structural comparison of the plant UGT homolog UGT71G1 with the predicted structure of human homolog UGT1A1. The structure of UGT71G1 is in complex with UDP (tan, PDB#: 2ACW); UGT1A1 was predicted using the AI software AlphaFold2 (light blue; UniProt# P22309; AlphaFold Protein Structure Database# AF-P22309-F1). UGT1A1 has a helical transmembrane-spanning region that is cropped from the image.

UGTs in mammals or humans are membrane-bound enzymes of the endoplasmic reticulum lumen, and therefore they contain transmembrane-spanning regions or motifs. By contrast, plant UGTs like UGT71G1 float freely in the cytoplasm [4]. X-ray crystal structures have been solved for a range of plant UGTs, such as UGT71G1 [1], UGT78K6 [9], UGT76G1 [10], Bc7OUGT [11], PaGT2 [12], and UGT74AC2 [13]; these proteins have >25% homology and we found that they are structural homologs based on TM-align analysis [14]. Therefore, we used the structure of UGT71G1 as a representative plant UGT for comparing with human UGTs. On the other hand, due to the inherent difficulties of purifying and crystalizing membrane-bound proteins, no complete crystal models exist for UGT1A1 or any other mammalian UGTs [15]. However, existing models for UGT71G1 and other cytoplasmic UGTs have facilitated the creation of predictive models for UGT1A1, which may be used to compare structures and potential binding sites. The UGT71G1 crystal structure [3] and the UGT1A1 AlphaFold2-predicted structure [16] are superimposed as shown in Figure 3. The highest similarity is in the C-terminal region, which is presumed to be involved in the binding of the UDP-sugar donor, with greater divergence in their N-terminal domain, which normally binds the sugar acceptor [3]. Additionally, the closest human homolog to UGT71G1 is UGT3A1, which shares 25% homology. The AlphaFold2 prediction is also available for UGT3A1 (from UniProtKB; accession Q6NUS8). A structural comparison between UGT71G1 and UGT3A1 shows significant differences in surface loops and helices, but they still align well enough to show a common origin. The listed acceptor substrates are the flavonoids kaempherol and quercetin [17], which are also substrates of UGT71G1 [18].

The UGT71G1 residues Asn-361, Glu-381, Gln-382, and Trp-360 within its consensus region collaborate to recognize and bind to the glucose moiety of UDP-glucose [1]. The analysis of the structures of UGT71G1 and PaGT2 complexed with UDP-glucose or the analog UDP-2fluoro-glucose, respectively, also shows the importance of Thr-143 in binding to the 6′OH of the glucose moiety [12]. Plant UGTs primarily use UDP-glucose as a donor, but also recognize UGT-xylose, UDP-galactose, UDP-glucuronic acid, and UDP-rhamnose [4]. Its usage of UDP-glucose instead of another UDP sugar is at least partially determined by the last amino acid of its consensus sequence being a glutamine (Gln-382). In homologs that recognize UDP-galactose, this residue is replaced with histidine. However, the discrimination between UDP-glucose and UDP-galactose appears to be more complex than a simple switch of histidine and glutamine. One study that mutated histidine to glutamine in a UDP-galactose-specific UGT instead gave the ability to use both UGT-glucose and UGT-galactose as sugar donors, while the mutation of Gln to His in a UGT specific to UDP-glucose did not grant galactosyltransferase activity [19]. Another study performing a His-to-Gln mutation in human UGT8, which forms galactosylated bile acids, found that the mutation of His to Gln in this UGT also caused the ability to use either UDP-glucose or UDP-galactose as sugar donors [20]. Due to hydrogen bonding with the 6′ hydroxyl of UDP-glucose, Thr-143 is essential to the discrimination between UDP-glucose and UDP-glucoronic acid. Thr-143 was shown to be replaced by glycine at this position in UGT73P12 [21]. Although the structure of UGT73P12 was determined without the UDP-glucoronic acid substrate, the sugar-binding group of Thr-143 can be seen to be functionally replaced with the side-chain of Arg-32, which reaches over to sit at the same position as Thr-143 in UGT71G1. The positive guanidino group of arginine would then bind with the 6′ carboxylate group of UDP-glucuronic acid. The mutation Arg32Ser induced a near-complete preference for UDP-glucose over UDP-glucuronic acid, presumably due to a loss of this favorable salt-bridge between Arg-32 and the 6′ carboxylate.

The functionality of the consensus sequence is recognized in UGT1A1, where point mutations within this region have been attributed to Crigler–Najjar Syndrome, a metabolic disease resulting from reduced or absent UGT1A1 activity [22]. The catalytic residue of UGT71G1 is His-22, which deprotonates the OH group of an acceptor molecule; additionally, Asp-121 may stabilize the active site to enhance the catalysis of the reaction [1]. This enables the accepter to attack the C1′ carbon of the UDP-glucose from the opposite side of the sugar ring. His-39 of UGT1A1 is in a similar position to UGT71G1 His-22, and missense mutations of it are also associated with decreased enzyme functions [22].

As one role of UGTs in many organisms is to derivatize toxic molecules, each enzyme homolog can target a broad range of substrates. UGTs are crucial to the detoxification, regulation, and excretion of endogenous substrates and xenobiotics [23]. Glycosylation increases the water solubility and modulates the bioactivity of most compounds, thereby facilitating excretion and further metabolic processes. Depending on the species, UGTs can perform O-linked glycosylation on flavonoids, sterols, triterpenes, and other cyclic or acyclic compounds with mostly available hydroxyl, but in some cases also with carboxyl groups. UGTs are present in most plant tissues and glycosylate a variety of endogenous triterpenes and flavonoids, including plant hormones, major classes of plant secondary metabolites, and xenobiotics like herbicides [4]. The glycosylation of these compounds by plant UGTs also aids in their storage in vacuoles [3]. Accordingly, mammal UGTs are expressed in, and are most concentrated in, tissues of the liver, colon, and small intestine, corresponding to their role as catalysts in phase II metabolism (Uniprot P22309, 2023). The UGT1A and UGT2B subfamilies have vital roles in phenolic drug elimination, which are not limited to acetaminophen, SN38, morphine, and assorted cancer drugs like irinotecan [15].

UGTs with high amino acid similarity can have similar patterns of metabolic activity, such as the UGT1A family and its glucuronidation of isoflavones [24]. However, it is notable that the sequence homology overall does not directly indicate if two UGTs have similar substrates or vice versa, such as how UGT1A9 and UGT71G1 both glycosylate quercetin despite low homology [17,25]. Potentially, there can be a significant similarity in substrate specificity among human UGTs and homologs from other species. Further characterization of UGTs is required to provide new insights into this aspect.

**Figure 4:**
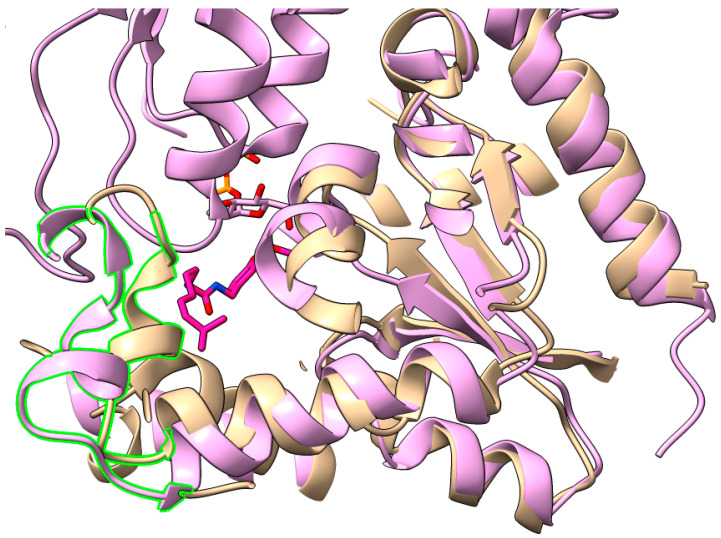
Size comparison of the acceptor-binding pocket of UGT71G1 with PaGT3. The highlighted helix of UGT71G1 (tan, PDB#: 2ACW) shows similarity in orientation with the highlighted portion of PaGT3 from *P. americana* (pink, PDB: 7VEL), relative to the capsaicin substrate of PaGT3 (dark pink). However, the helix in PaGT3 is positioned further away from the acceptor pocket, which corresponds to an increased pocket size in PaGT3 compared to UGT71G1, which allows binding to capsaicin.

**Figure 5:**
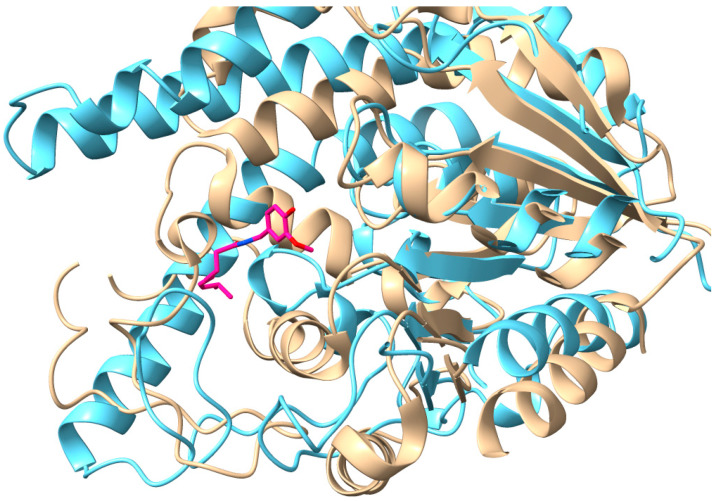
Size comparison of the acceptor-binding pocket of UGT71G1 with UGT1A1. The presumed acceptor-binding pocket of UGT1A1 (blue, UniProt ID: P22309) shows a greater volume compared to UGT71G1 (tan, PDB: 2ACW). Capsaicin (dark pink) is in the same location as in Figure 5, for reference.

New UGT structures with their acceptor substrates give insight into the important structural features of the N-terminal domain with regards to substrate specificity. In the recent structure of *Phytolacca americana* PaGT3 with substrates [26], an outward translation of the region homologous to Nβ5b to Nα5a can be seen (from the naming system used for describing UGT71G1). This opens up the substrate-binding pocket (Figure 4), allowing for binding of the longer, aliphatic substrate capsaicin as well as the more compact flavonoid kaempferol. Comparison with the UGT1A1 model prediction shows an even greater enlargement of this substrate cavity (Figure 5), which may relate to its broad substrate profile and allow for the glycosylation of relatively large substrates such as the antiviral drug raltegravir [27]. Comparing the PaGT3 structures with and without the acceptor substrate shows that the binding of the acceptor substrate does not induce a profound conformational change in the enzyme, implying that the opening of the acceptor-binding pocket is entirely due to differences in the enzyme sequence and folding and not dynamic changes in the protein during binding. In addition to the size of the active site pocket, it appears that the shape and hydrophobicity of the acceptor are important for PaGT3. The catalytic His-20 residue forms hydrogen bonds with the hydroxyl group, which is the target of the O-glycosylation. Most of the other surrounding residues in the active site have hydrophobic side-chains, especially phenylalanine and leucine, which surround the molecule on all sides. Kaempferol is a planar molecule, and thus kaempferol fits neatly into the active site. A bent substrate would likely be blocked from entering the active site through steric hindrance with the surrounding phenylalanine residues. PaGT3 will also glycosylate capsaicin, which is likely allowed by the flexibility of its aliphatic chain.

A greater understanding of acceptor-binding in UGTs raises the possibility of broadening their activity to include new substrates through mutagenesis. Thanks to structural information gleaned from the X-ray crystallography of rice UGT Os79 with an HT-2 mycotoxin, the specificity of Os79 could be expanded to include the related T-2 toxin, with the aim of improving the blight resistance of essential crop plants such as grain cereals, maize, and potato [28]. The T-2 toxin differs from the HT-2 toxin in only one functional group, with the T-2 toxin containing a larger acetyl group at the C4 carbon, which blocks binding with the enzyme. Three mutations, H122A/L123A/Q202L, expand the volume of the binding pocket and create an enzyme that deactivates the T-2 toxin and retains activity for other mycotoxins. The mutagenesis of plant UGTs is eased by the feasibility of expression in *E. coli* strains like BL21(DE3), which allows for the purification and testing of UGT mutants. The formation of glycosylated products can be verified using HPLC [1,9] or mass spectrometry [28].

## 4. Reactions of Plant and Human UGTs

While UGT homologs may have an overlapped spectrum of substrates, products can be substantially different, as each enzyme transfers a different sugar moiety to the substrate, which leads to orthogonal metabolic pathways. Generally, plant UGTs mainly use UDP-glucose as a co-substrate to perform glycosylation:

UDP-glucose + substrate → UDP + substrate-3-O-β-D-glucoside

Flavonoids and triterpenes are natural plant metabolites that have several functions in both plants and animals, notably as antioxidants [1]. Quercetin is a 7-hydroxyflavonol and phytoestrogen abundant in edible plants and is glycosylated by both plant and animal UGTs [17,25]. UGT71G1 produces five different quercetin glycosides with the majority being quercetin-3-O-glucoside, which is also the main glycoside formed by UGT78G1 and UGT85H2 [3]. Quercetin-3-O-glucoside, like other flavonols, has improved stability in plant tissues, but remains an effective antioxidant serving a protective role in photo-induced oxidative stress [3,29]. Chloroplasts are protected by antioxidant glycosides, which scavenge oxygen singlets and other free radicals produced by long-term white light exposure [30,31]. In addition, UGT84A1 and several other Arabidopsis UGT transcription factors are found to be up-regulated by light exposure and the light stress-indicative waste product H_2_O_2_ [32]. The production of flavonoid-generating enzymes is also upregulated in these conditions, indicating that UGTs have significance in flavonoid regulation. More recently, it was found that silencing UGT genes in Populus tremula x P. alba (poplar) led to decreased flavonoid concentrations and increased lipid peroxidation, further demonstrating that glycosylation by UGTs is an important regulatory step in flavonoid biosynthesis [33].

Previous studies demonstrated that UGT71G1 has a broad range of substrates. Although recombinant UGT71G1 glycosylates quercetin with high efficiency in vitro, the main natural substrate of UGT71G1 in *Medicago truncatula* is determined to be medicagenic acid, which is glycosylated into a triterpene saponin [25]. Triterpene saponins exhibit higher bioactivity than their aglycones and are potent antifungal and antimicrobial agents, as well as herbivory deterrents [34]. In a comparison of fungus-infested leaf tissue to non-infested leaf tissue, the concentration of medicagenic acid and UGT71G1 in *M. truncatula* was increased in the infested leaves [25]. UGT71G1 was generally found in all tissues but was most concentrated in leaves and flowers, which are major sites for infestation. Both the protective roles of saponins and flavonoid glycosides showcase the significance of UGT activity to plant health and defense.

Differing from plant UGTs, human UGTs mostly use UDP-glucuronic acid as the sugar moiety donor, instead of UDP-glucose, which leads to the following general reaction:

UDP-glucuronic acid + substrate → UDP + substrate-β-D-glucuronoside

Glucuronidation by UGTs accounts for a significant portion of metabolic pathways in humans, where the transfer of glucuronic acid to metabolites allows for further degradation and excretion through urine and bile [35]. Like in plants, human UGTs are often promiscuous and multiple species target the same compounds, though some have more specific functionalities. UGT1A1 is one of nine functional enzyme isoforms from the human UGT1A locus, yet it is the only significant catalyst of bilirubin glucuronidation [36]. Bilirubin is a degradation product of hemoglobin that requires multiple metabolic reactions to be eliminated. It is transformed into bilirubin monoglucuronide and then diglucuronide by UGT1A1, greatly increasing its water solubility for excretion (CID 5280352). This functionality is how phenobarbital, a drug that enhances the expression of UGT1A1 in the liver, was historically used as an effective treatment for hyperbilirubinemia [37].

The overlap in functionality of UGTs has been explored in studies by comparing the glucuronidation of a substrate class, such as endogenous steroids, and their transformation by UGT1As and UGT2Bs [38]. UGT1A1 most often conjugates estradiol and estrone at position 3 (3G), though it produces both 3G and 4G glucuronides of 4-OH estrone and 4-OH estradiol. 1A3 has similar but broader functionality. Most UGT isoforms produce negligible 2G products of 2-OH estrone and estradiol except for UGT1A8 and UGT1A9, which along with UGT2B7 form substantial amounts of 4G hydroxy estrogens [38]. The glucuronidation of androgens is primarily carried out by UGT2B15 and UGT2B17 [39]. Human UGT1A3 and UGT1A9 both glycosylate quercetin along with its derivative isorhamnetin [17]. Other phytoestrogens metabolized by human UGTs are the isoflavones genistein, daidzein, glycitein, biochanin A, and prunetin, which are metabolized by UGT1A1 in both the liver and intestines [24].

**Figure 6:**
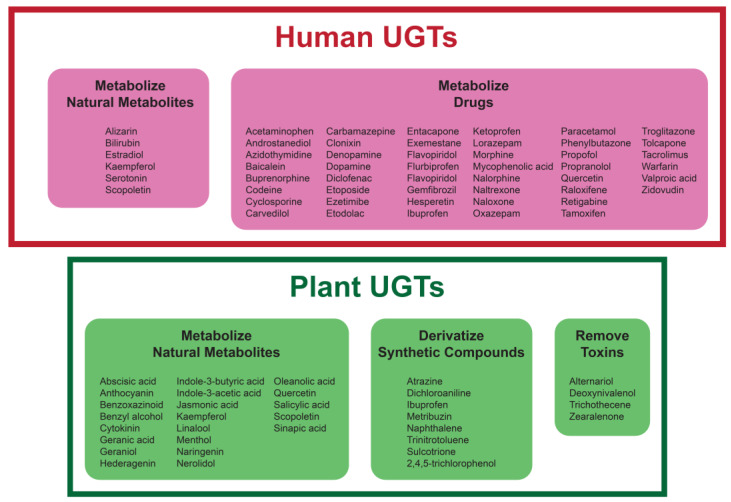
Representative examples of substrates for plant and human UGTs. These substrates include endogenous metabolite and synthetic molecules.

With these chemical activities, the roles of both plant and human UGTs include metabolizing endogenous metabolites and waste molecules and derivatizing exogenous compounds to reduce their toxicity [40,41]. Selected examples of UGT substrates for this biological functions are shown in Figure 6.

## 5. UGT1A1 Diseases Related to Dysfunctions of UGTs

As UGTs have essential roles in the metabolism, mutations among these enzyme genes lead to a range of genetic diseases. Some of these common diseases are described, followed by a discussion of the potential of using exogenous UGT homologs to target these health problems.

The loss of UGT1A1 activities leads to a range of diseases. UGT1A1 is the most significant isoform of UGT1A in the glucuronidation of bilirubin. Mutations in the UGT1A1 locus therefore can cause several diseases associated with hyperbilirubinemia or the build-up of bilirubin [22]. Gilbert syndrome is one type of hyperbilirubinemia caused by insertion mutations in the TATA-box of the UGT1A1 promoter. These mutations decrease the binding affinity between the promoter and TATA-box binding proteins, therefore decreasing UGT1A1 promoter activity [22,42]. Patients with Gilbert syndrome generally have normal liver function but may present a history of jaundice, along with non-specific symptoms like body aches and nausea [43]. While the condition does not require treatment nor pose a significantly increased risk of drug toxicity, patients with a combination of UGT1A SNPs are more likely to develop hyperbilirubinemia during treatment with atazanavir [44]. This protease inhibitor can inhibit the function of UGTs, though patients with SNPs of UGT1A1 alone are not conclusively at a greater risk for the side effects [45].

Contrasting with Gilbert syndrome are Crigler–Najjar syndromes, which are rare conditions characterized by the disrupted or absent function of the UGT1A1 enzyme due to assorted genetic mutations [22]. Crigler–Najjar syndromes are divided into type 1 (CN-1) and type 2 (CN-2), originally differentiated based on serum bilirubin levels and receptiveness to treatments [46]. Patients with CN-1 lack UGT1A1 activity entirely and often die within the first year of life from kernicterus or bilirubin build-up in the brain [47]. Phototherapy is the main treatment method, though a liver transplant is often the only viable treatment [47]. Patients with CN-2 have decreased UGT1A1 function but are receptive to more treatment methods. Historically, CN-2 has been treated with phenobarbital, which increases the production of their partially active UGT1A1, thereby lowering bilirubin levels [48]. Gene therapies for CN have recently reached clinical trials, and they use adeno-associated virus vectors to transfect UGT1A1 into patients [49]. Patients sustained normal bilirubin levels for 16 weeks in the lower-dosage group and 78 weeks for patients with a higher dosage.

Additionally, the loss of UGT activities may also affect the regulation of steroid levels. Glucuronidation by UGTs regulates steroid hormone levels and activity by enhancing their excretion and inhibiting steroids from binding with their receptors [50]. Abnormal signaling of these receptors, particularly estrogen receptor alpha and androgen receptor, is associated with hormone-dependent cancers [39]. Estrogens and androgens are transformed by several enzymatic pathways, but glucuronidation by UGTs is distinctly irreversible. UGTs are active and present in hormone-receptive tissues such as breast, prostate, and uterine tissues, along with steroid-glucuronates. The heightened expression of steroid-specific UGTs is associated with decreased steroid receptor activity, but steroid receptor activity can also affect UGT expression [39].

As mentioned earlier, UGTs 1A1, 1A3, 1A8, and 2B7 have high catalytic activities for estrogens and estrogen derivatives. Testosterone and DHT, which are potent ligands of the androgen receptor, are glucuronated by UGT2B15 and 2B17 [39]. Estradiol (E2) is the strongest natural ligand of ERα and can reversibly be converted to estrone (E1) by HSD17B1. Testosterone is directly converted to E2 by steroid aromatase, and thus both ERα and AR activity are closely intertwined with UGT activity. Significant derivatives of these steroids are catechol estrogens (CEs), which are hydroxylated E1 and E2 formed naturally through hepatic metabolism and within hormone-receptive tissues [38]. If CEs are not conjugated for removal, they can be oxidated into reactive semiquinones and quinones. Some of these final products are reactive with DNA and can cause cell mutations, which can eventually lead to cancer [51]. O-methylation by catechol-O-methyltransferase (COMT) is the most common pathway for CE elimination, but glucuronidation by UGT is an irreversible transformation that prevents further oxidation.

Furthermore, UGT dysfunctions often perturb the metabolism and the elimination of xenobiotics. The glucuronidation of drugs and other xenobiotics is a critical aspect of phase II metabolism and is enhanced with the presence of several UGTs binding to the same xenobiotic in life forms. The common analgesic morphine is glucuronated by several UGTs including 1A1, 1A8, and 2B7 [52]. All create morphine-6-glucuronates, which are stronger analgesics than morphine, though they lose activity quickly [53]. The painkiller acetaminophen is glucuronidated in the human liver by UGT1A6, 1A1, and 1A9 at varying affinities [54]. It was also noted that 1A6 was most active at lower acetaminophen levels, and 1A1 was most active at toxic concentrations. This concentration-dependent functionality denotes another benefit of redundant UGT specificities.

Many xenobiotics can alter the efficacy of other drugs or endogenous compounds, which can have negative effects even if the xenobiotic has known health benefits. Quercetin, like other phytoestrogens, interacts with estrogen receptors and their metabolism in animals by competing with hydroxyestradiols for COMT binding. In estradiol-treated animal models, quercetin administration decreased the excretion of methoxy-estradiols [55]. This competitive or noncompetitive inhibition is of health concern when regarding toxins such as bisphenol-A (BPA). BPA is a widely used manufacturing component of plastics and epoxy resin and a synthetic endocrine disruptor that competitively binds to estrogen receptors [56]. Higher BPA serum concentrations have been associated with polycystic ovary syndrome in young women and fetal malformations [57,58]. Glucuronidation by UGTs is the primary means of BPA elimination, and the resulting BPA-glucuronate is unable to bind with estrogen receptors [59]. In human liver microsomes, the recombinant UGT with the highest activity towards BPA was found to be UGT2B15, and to a lesser extent 2B7, 2B4, and 1B9 [60]. However, BPA was found to be an inhibitor of UGTs, particularly UGT2B isoforms [56]. The complete inhibition found for 2B4, 2B15, and 2B17 showcases that the BPA activity with UGTs found in earlier studies is not necessarily productive.

## 6. Potential of Exogenous UGT Homologs for Medical Applications

The dysfunction of UGTs leads to the accumulation of undesirable metabolites and xenobiotics that cause various health problems; these issues can be solved by elevating UGT activities. However, providing human UGT enzymes may not be practical, as these homologs are membrane proteins, and they are only functional in highly specific conditions. By contrast, a range of exogenous UGTs, such as the plant UGT71G1 that we discussed above, are cytosolic enzymes, and studies show that they are active even when expressed in bacteria.

Potentially, some of these UGT homologs can be used to reduce levels of disease-causing molecules, especially because these UGTs accept a broad spectrum of substrates and many of these homologs overlap in their substrate profile. Some of these enzymes derivatize health-promoting molecules and that may improve their solubility and thus increase their levels in human body. For instance, UGT75C1 from *Solanum lycopersicum* [61] and UGT75B1 from *Arabidopsis thaliana* [62] glycosylate abscisic acid, and this plant-based molecule stimulates the glucose uptake of various human tissues and organs, regulating glucose homeostasis [63,64]; UGT74F1 and UGT76B1 from *A. thaliana* [65] and UGT74J1 from *Oryza sativa* [66] metabolize salicylic acid, which has multiple targets in humans to provide beneficial effects, such as being anti-inflammation, anticancer, and providing neuroprotection [67]. Another two UGT homologs, UGT76E11 from *A. thaliana* [68] and UGT83A1 from *O. sativa* [69], are active towards several substrates, such as naringenin, quercetin, and kaempferol, in which naringenin is an antioxidant that improves human health via its antitumor, antiviral, antibacterial, anti-inflammatory, antiadipogenic, and cardioprotective effects [70]. On the other hand, some UGT homologs may protect humans by metabolizing toxic compounds, such as pollutants in the environment and toxins from food and pharmaceuticals. As an example, UGT73C5 from *A. thaliana* [71], Bradi5gUGT0330 from *Brachypodium distachyon* [72], and UGT12887 from *Tricum aestivum* [73] metabolize deoxynivalenol; this compound is a mycotoxin that is commonly present in rotten grains, such as corn, wheat, and rice, causing nausea, vomiting, diarrhea, abdominal pain, headache, and fever [74,75]. Another toxin, zearalenone, can be glycosylated by UGT73C5 and UGT73C6 from *A. thaliana* [76]; this toxin is known for disrupting the hormone balance in humans, which induces a range of diseases, including prostate, ovarian, cervical, and breast cancers [77]. These examples demonstrate that UGT homologs derivatize a wide range of targets that are relevant to human health and diseases.

While enzymatic activities from UGTs have a great potential for health-related applications, the direct use of UGT homologs for developing living therapeutics is rarely explored. By contrast, there are many studies of harnessing UGT genes to develop genetically modified organisms for potential agricultural applications, showing that it is highly feasible to use UGT superfamily genes for genetic engineering. Expressing some UGT homologs can provide crop protection, such as increasing drought tolerance by using the expression of UGT75B1 from *A. thaliana* [62], UFGT2 from *Zea mays*, and UGT74E2 from *A. thaliana* [78]; tolerance toward various infections can be improved by expressing UGT76D1 [79] and UGT84A2 [80] from *A. thaliana*, UGT12887 from *Tricum aestivum* [73], and Twi1 from *Solanum lycopersicum* [81]; and cold tolerance can be enhanced by expressing UGT90A1 from *O. sativa* [82] and UGT79B2 and UGT79B3 from *A. thaliana* [83]. Additionally, UGTs can potentially be harnessed for the biosynthesis of valuable molecular products; generally, many drugs are phenolic compounds and UGTs can derivatize them to create pharmaceutical libraries. For example, some UGT homologs were used to develop engineered yeast cells for generating triterpenoid compounds [84,85,86], which can be used to synthesize steroid-based drugs [87].

As discussed above, UGTs can catalyze a broad range of reactions that benefit human health, and thus they may be harnessed to engineer living therapeutics from bacteria and other types of cells. This is an emerging approach for disease prevention, diagnosis, and treatment. An unprecedented advantage of living therapeutics is their capability to sense and respond to changes in the host environment, such as the rise of a pathological trait, generated by the target disease. By implementing exogenous genes into the engineered cells, these living therapeutics gain new functions, such as monitoring the level of target biological and chemical entities, synthesizing drug molecules, and degrading toxins and other harmful substances. In recent years, this approach has emerged and has been used to explore a cure for a genetic disease, phenylketonuria [88]. Probiotics were also genetically modified to detect infectious diseases and inhibit the growth of corresponding pathogens [89,90,91,92]. Some studies have also built engineered probiotics to generate hormone-like molecules that may control inflammatory bowel disease, diabetes, and obesity [93,94,95,96]. Together, these studies demonstrate that living therapeutics is a promising strategy for drug development. 

While a large portion of living therapeutics research focuses on delivering engineered cells to the gastrointestinal tract, several studies have shown that a microbiome approach can be used to deliver bacteria to the liver for implementing engineered biological functions [97,98,99]. It is likely that some species of microbes from the gut microbiome robustly translocate to the liver. By engineering these microbes to gain UGT activities, they can be used to metabolize target disease-causing metabolites and xenobiotics in the liver, reducing levels of these molecules. Delivering exogenous UGTs to the liver can be beneficial, as it is the main organ for performing UGT activities. Potentially, after derivatization by exogenous UGTs, those products may also be excreted via bile to the gut for removal from the body.

## 7. Conclusions

Despite differences in structure and amino acid sequences, even distant plant and animal UGTs may share substrate specificities and hold similar metabolic functions. As described in this review article, numerous UGT genes have been isolated and expressed in new hosts for various applications in agriculture, metabolic engineering, and drug discovery. A large number of UGTs have been and continue to be discovered, and there is a huge potential for further harnessing these UGT enzymes for tackling problems in our society.
